# Effect of time of administration on cholesterol-lowering by psyllium: a randomized cross-over study in normocholesterolemic or slightly hypercholesterolemic subjects

**DOI:** 10.1186/1475-2891-3-17

**Published:** 2004-09-28

**Authors:** Guido MA Van Rosendaal, Eldon A Shaffer, Alun L Edwards, Rollin Brant

**Affiliations:** 1 Department of Community Health Sciences, University of Calgary, 3330 Hospital Drive NW, Calgary, Alberta, Canada, T2N 4N1; 2 Department of Medicine, University of Calgary, 3330 Hospital Drive NW, Calgary, Alberta, Canada, T2N 4N1

## Abstract

**Background:**

Reports of the use of psyllium, largely in hypercholesterolemic men, have suggested that it lowers serum cholesterol as a result of the binding of bile acids in the intestinal lumen. Widespread advertisements have claimed an association between the use of soluble fibre from psyllium seed husk and a reduced risk of coronary heart disease. Given the purported mechanism of cholesterol-lowering by psyllium, we hypothesized that there would be a greater effect when psyllium is taken with breakfast than when taken at bedtime. Secondarily, we expected to confirm a cholesterol-lowering effect of psyllium in subjects with "average" cholesterol levels.

**Methods:**

Sixteen men and 47 women ranging in age from 18 to 77 years [mean 53 +/- 13] with LDL cholesterol levels that were normal or slightly elevated but acceptable for subjects at low risk of coronary artery disease were recruited from general gastroenterology and low risk lipid clinics. Following a one month dietary stabilization period, they received an average daily dose of 12.7 g of psyllium hydrophilic mucilloid, in randomized order, for 8 weeks in the morning and 8 weeks in the evening. Change from baseline was determined for serum total cholesterol, LDL, HDL and triglycerides.

**Results:**

Total cholesterol for the "AM first" group at baseline, 8 and 16 weeks was 5.76, 5.77 and 5.80 mmol/L and for the "PM first" group the corresponding values were 5.47, 5.61 and 5.57 mmol/L. No effect on any lipid parameter was demonstrated for the group as a whole or in any sub-group analysis.

**Conclusion:**

The timing of psyllium administration had no effect on cholesterol-lowering and, in fact, no cholesterol-lowering was observed. Conclusions regarding the effectiveness of psyllium for the prevention of heart disease in the population at large may be premature.

## Background

A cholesterol lowering effect has been reported for a variety of soluble dietary fibres [1-5]. In February, 1998, the U.S. Food and Drug Administration authorized the use, on food labels and food labelling, of health claims on the association between soluble fibre from psyllium seed husk and a reduced risk of coronary heart disease [6]. Among the suggested mechanisms by which soluble fibre lowers cholesterol is the binding of bile acids in the intestinal lumen resulting in decreased absorption and increased faecal excretion of them [7-15]. The ensuing bile acid depletion increases hepatic demand for the de novo synthesis of bile acids from cholesterol. This requirement is met, in part, by increased hepatic LDL receptor activity, which in turn reduces circulating LDL.

The accumulation and concentration of bile in the gallbladder is a continuous process. In rats which lack a gallbladder, the biliary excretion rate of bile salts is maximal at night [16] and bile is stored in the gallbladder during an overnight fast. To the extent that the cholesterol-lowering effect of psyllium requires an interaction with bile, the magnitude of its cholesterol-lowering effect should vary with the quantity of bile in contact with a given amount of psyllium. The gallbladder empties with a meal and the total quantity of bile salt presented to the small bowel should be highest when this emptying occurs at breakfast, following a fast and the associated overnight accumulation of secreted bile. Conversely, psyllium presented to the gut following a short fast and without the full stimulus to gallbladder emptying of an associated meal should result in an encounter with a smaller amount of bile. We accordingly set out to test the hypothesis that administering psyllium with breakfast would have a significantly greater cholesterol-lowering effect than would taking a similar dose at bedtime. Since the predominance of the literature with regard to the cholesterol-lowering effect of psyllium is in individuals with hypercholesterolemia, a secondary goal of our study was to confirm the cholesterol-lowering effect of psyllium in subjects with "average" cholesterol levels.

## Methods

Patients identified in gastroenterology practices as requiring long-term treatment with psyllium, typically for chronic constipation or the irritable bowel syndrome, were invited to participate in the study. In addition, individuals who had received dietary counselling in a lipid clinic regarding cholesterol-lowering and who subsequently had cholesterol levels deemed not to require further intervention because they met targets set out in clinical practice guidelines were invited by clinic staff to participate. Subjects were deemed ineligible if they were under age 18 years, were under active treatment for hyperlipidemia, had total cholesterol greater than 7.00 mmol/L, required alterations in dosage of medications which might have an effect on lipid levels, had had a gastrectomy, had any disease which is associated with hyperlipidemia, were receiving a bile acid binding resin, or if they did not eat breakfast regularly.

The study was approved by the Conjoint Health Research Ethics Board of the University of Calgary, Faculty of Medicine. Subjects were given a description of the study indicating our interest in comparing the relative efficacy of hs versus am dosing with psyllium without indicating the specific hypothesis, and were asked to sign a consent form.

Gastroenterology patients were given a high fibre diet sheet as part of their therapeutic regimen and were asked to take this on a continuing basis, beginning one month prior to the initiation of the study. The diet sheet emphasized dietary sources containing predominantly insoluble fibre. Lipid clinic patients had all received in-depth counselling regarding dietary measures for hypercholesterolemia, including a high fibre regimen, and had implemented their dietary changes at least one month before beginning the study. An unsweetened psyllium preparation, "Novo-Mucilax" [NovoPharm], providing three grams of hydrophilic mucilloid per 6.2 gram powder, and a scoop known to provide at least ten grams of psyllium were provided. Containers were numbered and weighed at the conclusion of each test interval. Subjects were randomized to initially take a scoop full of psyllium either with breakfast or at bedtime. Using a crossover design, psyllium was taken in the morning or evening for eight weeks and at the alternate time for the subsequent eight weeks.

Determinations of serum total cholesterol, LDL, HDL and triglycerides were made before beginning psyllium, at eight weeks and at sixteen weeks after commencing its use. A trained dietician obtained a dietary history and patients were weighed at the beginning and at the conclusion of the study. All lipid determinations were undertaken following a 14 hour fast and analyses were done in a central laboratory.

After initial data inspection based on boxplots and summary measures, cholesterol values at 8 and 16 weeks were examined using analysis of variance, taking into account treatment, period, and between and within subject effects in accordance with the cross-over design. The pattern of change from baseline to 16 weeks was evaluated using paired t-tests.

## Results

Of 86 subjects beginning the study, 33 of those referred from the gastroenterology clinics and 30 of those referred from the lipid clinic completed it. Of those withdrawing, eight did so because they could not tolerate the psyllium or it was felt to interfere with prescribed medication, 2 had elevated lipids, 4 did not complete all the required blood work, 5 were unable to comply with the protocol because of work or lifestyle changes and 4 developed intercurrent diseases which precluded completing the protocol.

The age range of subjects was 18 to 77 years, mean 53 +/- 13 years, including 16 men and 47 women. The mean dose of psyllium taken was 12.7 +/ - 2.3 g for morning dosing and 12.7 +/- 2.2 g when taken in the evenings.

Values for total, LDL, HDL cholesterol and triglycerides at baseline, eight and sixteen weeks for various subgroupings are tabulated in tables 1, 2, 3 and 4, respectively. Data for changes from baseline for total, LDL and HDL cholesterol and triglycerides are listed in table 5. Changes from baseline were not significant for any parameter. Furthermore, the confidence intervals given in Table 5 show that this data essentially excludes a clinically significant psyllium effect.

**Table 1:** Total Cholesterol in mmol/L [SD]

**[n]**	**Baseline**	**8 wks**	**16 wks**
AM first	5.76 [0.94]	5.77 [1.01]	5.80 [0.95]
PM first	5.47 [0.98]	5.61 [1.10]	5.57 [1.06]
Females [47]	5.72 [0.96]	5.79 [1.07]	5.74 [0.99]
Males [16]	5.29 [0.94]	5.39 [0.96]	5.48 [1.04]
GI subjects [33]	5.00 [0.83]	5.06 [0.91]	5.07 [0.84]
Lipid clinic Subjects [30]	6.28 [0.59]	6.39 [0.69]	6.35 [0.70]
All subjects [63]	5.61 [0.96]	5.69 [1.05]	5.68 [1.00]

"AM first" are subjects taking psyllium in the morning for the first 8 weeks and in the evening for the second 8 weeks. Vice versa for "PM first".

**Table 2:** LDL CHOLESTEROL in mmol/L [SD]

	**Baseline**	**8 wks**	**16 wks**
AM first	3.55 [1.01]	3.51 [1.04]	3.52 [0.94]
PM first	3.39 [0.90]	3.43 [1.03]	3.45 [0.92]
Females	3.55 [0.92]	3.54 [1.04]	3.52 [0.92]
Males	3.22 [1.03]	3.24 [0.99]	3.39 [0.97]
GI subjects	2.89 [0.81]	2.85 [0.85]	2.91 [0.72]
Lipid clinic subjects	4.09 [0.66]	4.15 [0.72]	4.11 [0.69]
All subjects	3.47 [0.95]	3.47 [1.03]	3.48 [0.92]

**Table 3:** HDL CHOLESTEROL in mmol/L [SD]

	**Baseline**	**8 wks**	**16 wks**
AM first	1.38 [0.29]	1.42 [0.37]	1.42 [0.33]
PM first	1.38 [0.40]	1.39 [0.36]	1.36 [0.33]
Females	1.41 [0.30]	1.44 [0.30]	1.42 [0.29]
Males	1.28 [0.45]	1.29 [0.50]	1.30 [0.43]
GI subjects	1.36 [0.39]	1.41 [0.43]	1.37 [0.38]
Lipid clinic subjects	1.40 [0.30]	1.40 [0.28]	1.41 [0.27]
All subjects	1.38 [0.35]	1.41 [0.36]	1.39 [0.33]

**Table 4:** TRIGLYCERIDES in mmol/L [SD]

	**Baseline**	**8 wks**	**16 wks**
AM first	1.81 [0.70]	1.84 [0.86]	1.86 [0.91]
PM first	1.53 [0.52]	1.69 [0.70]	1.65 [0.61]
Females	1.66 [0.61]	1.72 [0.70]	1.76 [0.71
Males	1.70 [0.68]	1.89 [0.99]	1.72 [0.94]
GI subjects	1.63 [0.54]	1.71 [0.82]	1.70 [0.72]
Lipid clinic subjects	1.71 [0.71]	1.82 [0.74]	1.80 [0.83]
All subjects	1.67 [0.35]	1.76 [0.78]	1.75 [0.77]

**Table 5:** Mean changes from baseline at 8 and 16 weeks [mmol/L] and 95% confidence intervals [all patients, n = 63]

	**8 wks**		**16 wks**	
TOTAL	0.080	p = 0.26	0.068	p = 0.28
CHOLESTEROL	[-0.061,0.22]		[-0.056,0.19]	
LDL	0.002	p = 0.98	0.018	p = 0.75
CHOLESTEROL	[-0.13,0.14]		[-0.098,0.13]	
HDL	0.028	p = 0.28	0.012	p = 0.61
CHOLESTEROL	[-0.023,0.08]		[-0.035,0.059]	
TRIGLYCERIDES	0.094	p = 0.22	0.082	p = 0.22
	[-0.059,0.25]		[-0.050,0.21]	

The mean caloric intake or the intakes of fat or fibre did not change significantly during the study [table 6]. There was a small but significant increase in the weights of subjects, which was felt to reflect the high proportion of subjects beginning the study in the fall and the associated reduction of physical activity during the subsequent winter months. There was no apparent relationship between change in cholesterol and change in weight [correlation = .1, p = .15].

**Table 6:** Weight and Dietary Intake [SD] at Baseline and at 16 Weeks

	**Baseline**	**16 wks**	
WEIGHT [Kg]	74.6 [16.5]	75.4 [16.9]	p = 0.0039
ENERGY [kcal]	1649 [409]	1689 [419]	p = 0.29
FAT [g]	49.1 [20.3]	49.8 [20.2]	p = 0.66
FIBER [g]	19.7 [7.96]	19.7 [7.28]	p = 0.99

## Discussion

We failed to prove our hypothesis that administration of psyllium in the morning would have a greater cholesterol-lowering effect than it would in the evening. Not only was there no observable difference in lipid levels between the crossover periods but the daily ingestion of a greater daily dose than the 10.2 g of psyllium for which the FDA allows health claims to be made [6] had no effect on lipid levels in our study group. No change in any lipid parameter, including total and LDL cholesterol was observed. No difference was found when subgroup analysis was undertaken for the sex of the patients, the time of day they took their psyllium, or whether they were recruited from the gastroenterology clinic or the lipid clinic.

We used a crossover design since this was the most appropriate one for the primary question being addressed; accordingly, our study did not include a control group. However, the nature of the study should not have provided any motivation for study subjects to adopt any new lifestyle or dietary changes beyond those implemented well before the introduction of psyllium. Observational data has been shown to provide valid information, which is consistent with that observed in randomized, controlled trials [17,18]. The nature of the intervention was in keeping with those undertaken in day-to-day clinical practice and the protocol used should, therefore, have high "clinical relevance".

Failure of lipid-lowering by psyllium has also been demonstrated in twenty hypercholesterolemic children [19], in twenty-four hyperlipidemic adults [20] and in a large observational study of elderly patients taking psyllium [21]. A report of lipid-lowering therapies in hypercholesterolemic veterans showed only a 2% reduction in LDL cholesterol and a small increase in LDL/HDL ratio in patients taking psyllium, but does not provide a measure of statistical significance [22]. One study revealed no difference in total cholesterol-lowering compared to placebo, but a reduction of LDL cholesterol resulted from psyllium treatment [23]. Another demonstrated no difference in total cholesterol-lowering compared to placebo, a reduction of LDL in 11 "responders" and no change in 9 "nonresponders" [24]. A reduction of HDL cholesterol has been noted in some studies [25-27] and was associated with changes in LDL/HDL ratios similar to placebo treatment [25,26].

Published studies include few normocholesterolemic subjects. Cholesterol reduction was observed in 7 normal men [28] and in 5 of 9 subjects [29], in both studies after 3 weeks of treatment. A reduction of cholesterol levels was also observed in 12 elderly patients given psyllium for 4 months [30], while 5 normocholesterolemic subjects in another study showed no reduction after 2 to 7 months of treatment [31].

A meta-analysis of 17 studies of patients with hypercholesterolemia has suggested a small but significant cholesterol-lowering effect of psyllium [2]. All of these investigations were associated in one way or another with the product manufacturer. Additional studies have also indicated some cholesterol-lowering by psyllium in hypercholesterolemic individuals [32-37] or in diabetics [38-40]; however, much of this work is uncontrolled and some protocols have specifically excluded premenopausal women [33,38]. The association of cholesterol-lowering effects with psyllium may be weakened in some studies by the use of a supplement containing additional forms of soluble fibre [42] or by apparent differences in intake of calories [43-46], soluble fibre [25] or cholesterol [47] in control and treatment groups or periods. Several reports include only small numbers of patients and/or are of short duration. There is a strong predominance of male subjects in these publications and some protocols incorporate additional treatment interventions [20,48].

Several factors may contribute to the difference between our observations and those of others. A meta-analysis has demonstrated that the initial level of cholesterol was highly predictive of the subsequent reduction of cholesterol by oat bran [49]. A greater effect of psyllium in men compared to women has been suggested [23,46] and a diet high in soluble fibre produced less cholesterol-lowering in post menopausal women than in men [10]. Soluble fibre has a lesser effect on lipid metabolism in female than in male guinea pigs [50] and there is a sex-based difference in mechanism of action in this animal [51]. Oat bran fails to lower cholesterol in young women, in contrast to men and older women [52]. The dominance of women in our study, the "normal", or only slightly abnormal cholesterol states of our subjects and the relatively young ages of some of them may, accordingly, account for some of the variance of our observations with some of those previously reported.

The small increase in the weight of subjects is believed to be have resulted from reduced physical activity. In a meta-analysis of the effect of weight reduction on lipids, predominantly through dietary change, a reduction in total cholesterol of 0.05 mmol/L and of 0.02 mmol/L in LDL cholesterol per kilogram of weight lost was identified [53]. Dietary intakes were stable throughout our study and the average weight gain of less than one kilogram is very unlikely to have raised cholesterol levels to a degree sufficient to offset a significant cholesterol lowering effect of psyllium.

A small cholesterol-lowering effect of psyllium appears to occur in hypercholesterolemic individuals, at least in men and possibly postmenopausal women. The notion of a benefit accruing to the general population requires additional study. The promotion of foods containing psyllium as reducing the risk of heart disease for the population at large [6] may be premature. Additional study is required and this should be undertaken in a manner that is free from concern regarding the possibility of publication bias which Brown L, Rosner B, Willett WW and Sacks FM have raised [2].

## Conclusion

The timing of psyllium administration had no effect on cholesterol-lowering and, in fact, no cholesterol-lowering was observed. Conclusions regarding the effectiveness of psyllium for the prevention of heart disease in the population at large may be premature.

## Competing interests

The authors declare that they have no competing interests.

## Authors' contributions

GVR carried out the study design, data review and writing. EAS carried out the study design and data review. RB carried out the study design, data review and statistical analysis. ALE carried out the study design and data review.
